# Cofilin 1 promotes the pathogenicity and transmission of pathological α-synuclein in mouse models of Parkinson’s disease

**DOI:** 10.1038/s41531-021-00272-w

**Published:** 2022-01-10

**Authors:** Mingmin Yan, Min Xiong, Lijun Dai, Xingyu Zhang, Yunhong Zha, Xiaorong Deng, Zhui Yu, Zhentao Zhang

**Affiliations:** 1 Department of Neurology, Renmin Hospital of Wuhan University, Wuhan, 430060 China; 2 Department of Neurology, Hubei NO. 3 People’s Hospital of Jianghan University, Wuhan, 430060 China; 3 The People’s Hospital of China Three Gorges University, the First People’s Hospital of Yichang, Yichang, 443000 China; 4 Department of Critical Care Medicine, Renmin Hospital of Wuhan University, Wuhan, 430060 China

**Keywords:** Parkinson's disease, Parkinson's disease

## Abstract

The pathological hallmark of Parkinson’s disease (PD) is the presence of Lewy bodies (LBs) with aggregated α-synuclein being the major component. The abnormal α-synuclein aggregates transfer between cells, recruit endogenous α-synuclein into toxic LBs, and finally trigger neuronal injury. However, the molecular mechanisms mediating the aggregation and transmission of pathological α-synuclein remain unknown. Previously we found that cofilin 1, a member of the actin-binding protein, promotes the aggregation and pathogenicity of α-synuclein in vitro. Here we further investigated the effect of cofilin 1 in mouse models of PD. We found that the mixed fibrils composed of cofilin 1 and α-synuclein are more pathogenic to mice and more prone to propagation than pure α-synuclein fibrils. Overexpression of cofilin 1 enhances the seeding and spreading of α-synuclein aggregates, and induces PD-like behavioral impairments in mice. Together, these results illustrate the important role of cofilin 1 in the pathogenicity and transmission of α-synuclein during the onset and progression of PD.

## Introduction

The histopathological hallmark of Parkinson’s disease (PD) is the presence of intracellular aggregates referred to as Lewy bodies (LBs), in which α-synuclein is the major component. The aggregation of α-synuclein triggers the degeneration of dopaminergic neurons. Thus, the conversion of soluble α-synuclein into insoluble aggregates plays a key role in the pathogenesis of PD^1^. Misfolded α-synuclein acts as templates to change the conformation of soluble α-synuclein, leading to the formation of aggregates. Pathological studies found that α-synuclein pathology spreads in a stereotyped fashion in PD brain^2^. Interestingly, synthetic α-synuclein fibrils induce the aggregation of endogenous α-synuclein and neuronal injury in cultured neurons^3–5^. Furthermore, intrastriatal inoculation of α-synuclein fibrils induced the propagation of α-synuclein pathology and degeneration of dopaminergic neurons in mouse brain^6^. These observations provide the basis for the “prion hypothesis” of PD^7^.

Fibrillary α-synuclein aggregates are characteristic of synucleinopathies including PD, multiple system atrophy (MSA), dementia with Lewy bodies (DLB), and pure autonomic failure (PAF)^8^. It has been reported that α-synuclein aggregates derived from PD and MSA retain different conformation and seeding activity^9,10^. In vitro evidence showed that α-synuclein forms different fibrillar polymorphs under different conditions^11^. The structural variants of α-synuclein aggregates may account for their distinct biochemical and functional properties^12,13^. Vulnerable brain regions suffer from the α-synuclein pathology earlier. Conversely, neurons that resist the attack of α-synuclein pathology can survive by benefiting from cellular compensation^14^. Therefore, insights into the aggregation and propagation of α-synuclein are of great help in understanding the pathological progression of PD and other synucleinopathies. However, the molecular mechanisms underlying the formation of distinct α-synuclein strains and relevant regulatory mechanisms of their transmission remain unknown.

Cofilin is a member of actin-binding proteins expressed in all eukaryotes, regulating cytoskeleton dynamics so as to manipulate cell morphology, locomotion, migration, invasion, and cytokinesis, etc^15^. Cofilin 1 is the major isoform of cofilin that is expressed in the brain. Cofilin 1 plays a pivotal role in multiple neurodegenerative diseases. Quantitative proteomic analysis found that cofilin 1 is one of the various proteins located in LBs^16^. However, the roles of cofilin 1 in the pathogenesis of PD is currently unclear. In our previous study, we found that cofilin 1 binds α-synuclein and promotes its aggregation and transmission in vitro^17^. Here we further investigated the effect of cofilin 1 on the aggregation and spreading of α-synuclein pathology in vivo. We discovered that the mixed fibrils consist of cofilin 1 and α-synuclein are more toxic and prone to induce the spreading of α-synuclein pathology than pure α-synuclein fibrils in the mouse brain. Moreover, overexpression of cofilin 1 promotes the pathogenicity and transmission of α-synuclein aggregates and induces more severe dopaminergic degeneration and motor impairments in a mouse model of PD. All these results demonstrate the important role of cofilin 1 in the pathogenesis of PD.

## Results

### Mixed fibrils consist of cofilin 1 and α-synuclein are more pathogenic than pure α-synuclein fibrils in vivo

**Fig. 1:**
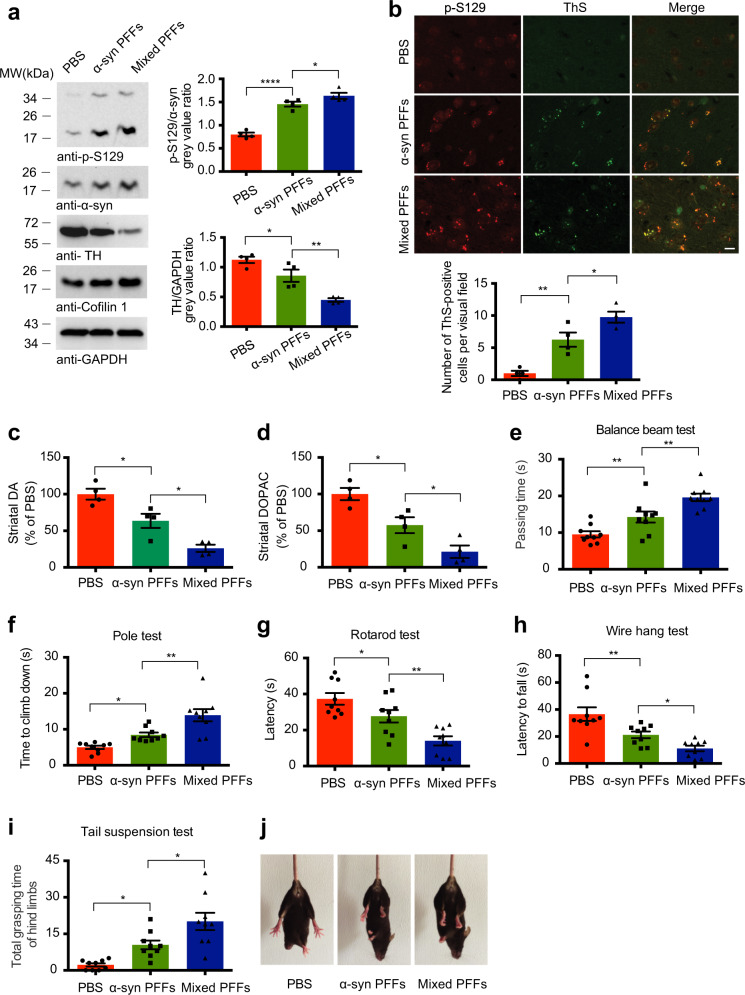
Mixed fibrils consist of cofilin 1 and α-synuclein are more pathogenic than pure α-synuclein fibrils in vivo. **a** Western blot analysis of TH, p-S129 α-synuclein and total α-synuclein in the striatum of the mouse brain. Data are mean ± SEM; *n* = 4; *P < 0.05, **P < 0.01, ****P < 0.0001 by one-way ANOVA. **b** Colocalization of p-S129 α-synuclein and thioflavin S staining in the right striatum. Scale bar, 20 μm. Data are mean ± SEM; *n* = 4; *P < 0.05, **P < 0.01 by one-way ANOVA. **c**, **d** Concentrations of DA and DOPAC in the striatal tissues, as determined by HPLC. Data are mean ± SEM; *n* = 4 mice per group; *P < 0.05 by one-way ANOVA. (**e**–**j**) Behavioral tests. The mixed PFFs induced more severe motor impairments, as demonstrated by balance beam test (**e**), pole test (**f**), rotarod test (**g**), wire hang test (**h**), and tail suspension test (**i**). Data are mean ± SEM; *n* = 9 mice per group; *P < 0.05, **P < 0.01 by one-way ANOVA. (**j**) Representative images showing the hindlimbs clasp in tail suspension test.

Previously we found that cofilin 1 binds α-synuclein and facilitates its aggregation. The cofilin 1-α-synuclein mixed fibrils are more toxic than pure α-synuclein fibrils in vitro^9^. To assess the composition of cofilin 1-α-synuclein mixed fibrils, we performed electron microscopy (EM) (Supplementary Fig. 1a, b) and immuno-EM (Supplementary Fig. 1c–f) with antibodies to α-synuclein and cofilin 1. Pure α-synuclein fibrils were labeled solely with α-synuclein antibody (Supplementary Fig. 1c, e), while cofilin 1-α-synuclein mixed fibrils were labeled with both α-synuclein and cofilin 1 antibodies (Supplementary Fig. 1d, f), suggesting that cofilin 1 and α-synuclein interact to form fibrils with mixed composition. To investigate the seeding activity of mixed fibrils in vivo. We used a well-established mouse model of synucleinopathy by injecting pure α-synuclein fibrils and mixed fibrils into the right striatum of wild-type mice, respectively^6^. As expected, intracranial injection of α-synuclein fibrils induced the phosphorylation and dimerization of endogenous α-synuclein at Ser129. The expression of tyrosine hydroxylase (TH), a marker of dopaminergic neurons in the striatum was decreased. These results indicate that α-synuclein fibrils induced both α-synuclein pathology and dopaminergic neuronal injury, two major pathological characterizes of PD. Interestingly, the mixed fibrils induced more p-Ser129 α-synuclein expression and less TH expression than pure α-synuclein fibrils, as indicated by Western blot (Fig. 1a; for p-S129, *n* = 4, F (2, 9) = 65.36, *P1* < 0.0001, *P*2 = 0.0445; for TH, *n* = 4, F (2, 9) = 23.86, *P1* = 0.0239, *P2* = 0.005). Moreover, p-Ser129 α-synuclein colocalized with thioflavin S (ThS) in the striatum, indicating that both α-synuclein fibrils and mixed fibrils induced the fibrillization of α-synuclein in the brain (Fig. 1b; *n* = 4, F (2, 9) = 27.38, *P1* = 0.0034, *P2* = 0.0165). HPLC assay found that the content of striatal dopamine (DA) and its metabolite DOPAC in mice injected with α-synuclein fibrils was decreased compared to the control group. The decrease of striatal DA and DOPAC in mice injected with the mixed fibrils was more severe than that in mice injected with pure α-synuclein fibrils (Fig. 1c; *n* = 4 mice per group, F (2, 9) = 23.7, *P* = 0.0135; Fig. 1d, *n* = 4 mice per group, F (2, 9) = 17.98, *P1* = 0.0204, *P2* = 0.0222). Furthermore, we assessed the behavioral impairments of mice in different groups, including the balance beam test, pole test, rotarod test, wire hang test, and tail suspension test. Interestingly, the mixed fibrils induced more severe motor deficits in mice than the α-synuclein fibrils (Fig. 1e–j, *n* = 9 mice per group; Fig. 1e, F (2, 24) = 19.07, *P1* = 0.0081, *P2* = 0.0062; Fig. 1f, F (2, 24) = 17.46, *P1* = 0.0321, *P2* = 0.003; Fig. 1g, F (2, 24) = 14.23, *P1* = 0.0377, *P2* = 0.0095; Fig. 1h, F (2, 24) = 13.82, *P1* = 0.0086, *P2* = 0.0496; Fig. 1i, F (2, 24) = 14.84, *P1* = 0.0196, *P2* = 0.0142). These results demonstrate that the cofilin 1-α-synuclein mixed fibrils are more pathogenic than pure α-synuclein fibrils to induce α-synuclein pathology in vivo.

### The mixed fibrils promote the spreading of α-synuclein pathology through the brain

**Fig. 2:**
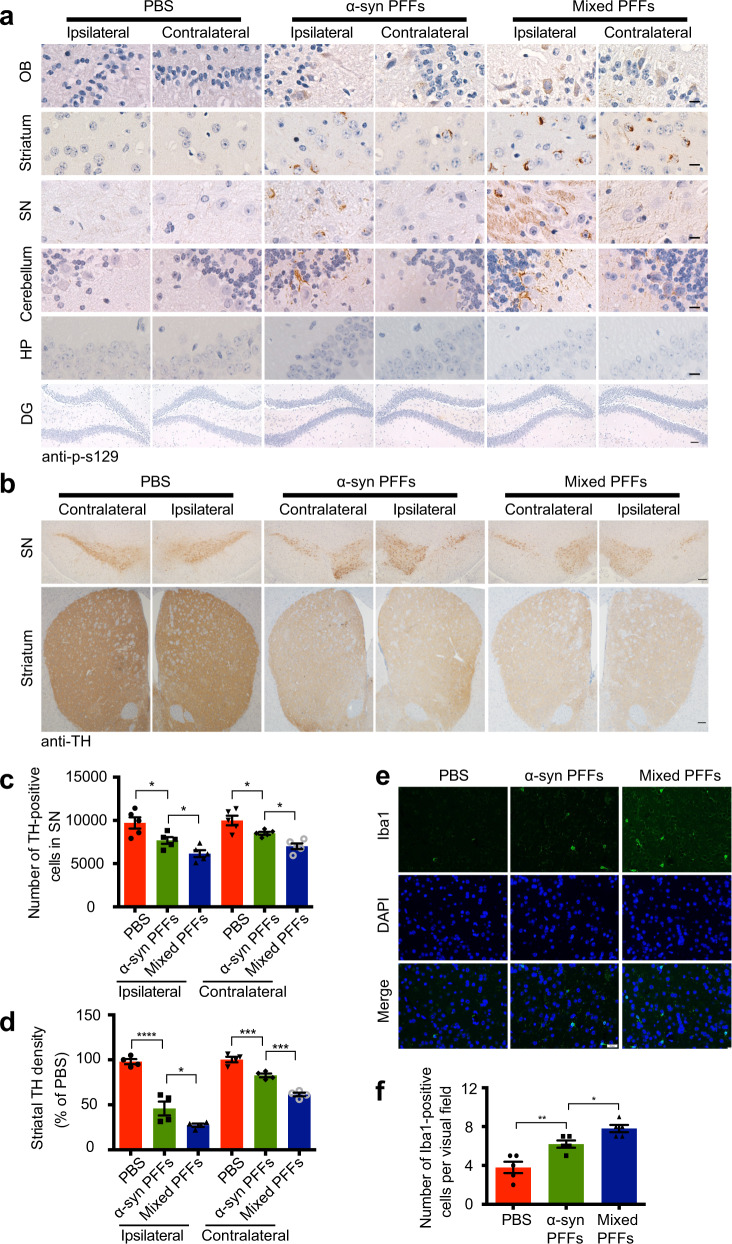
The mixed fibrils are more prone to transmission in mouse brain. **a** Immunohistochemistry showing the expression of p-S129 α-synuclein in different brain areas from mice injected with PBS, α-synuclein PFFs, and mixed PFFs. Scale bar in DG area, 50 μm. Scale bars in other areas, 20 μm. **b**–**d** Immunostaining of TH in the striatum and substantia nigra (SN). Unbiased stereological cell counts in the SN and total density of striatal dopaminergic terminals show that mixed fibrils induced more severe dopaminergic neuronal loss compared with pure α-synuclein fibrils. Scale bars, 100 μm. Bar graph, quantification of the TH-positive neurons in the SN and TH density in the striatum. Data are mean ± SEM; *n* = 4–5; *P < 0.05, ***P < 0.001, ****P < 0.0001 by one-way ANOVA. (**e**, **f**) Immunostaining showing the Iba1-positive microglia in the striatum of mice injected with PBS, α-synuclein PFFs, and mixed PFFs. Scale bar, 20 μm. Data are mean ± SEM; n = 5; *P < 0.05, **P < 0.01 by one-way ANOVA.

α-Synuclein pathology transmits between brain areas during the progression of PD. To illustrate the influence of α-synuclein fibrils and mixed fibrils on the transmission of α-synuclein pathology in mouse brain, we injected PBS, α-synuclein fibrils, and cofilin 1-α-synuclein mixed fibrils, respectively, into the right striatum of wild-type mice. 24 Weeks after injection, we assessed the transmission of α-synuclein pathology in different brain areas. Phosphorylation of endogenous α-synuclein was found in the olfactory bulb (OB), striatum, substantia nigra (SN), and cerebellum, indicating the transmission of the α-synuclein pathology. The hippocampus and dentate gyrus (DG) were not affected at this time point. As expected, the mixed fibrils were more potent to induce bilateral α-synuclein pathology than pure α-synuclein fibrils (Fig. 2a, Supplementary Fig. 2a, b; Supplementary Fig. 2a, *n* = 4, F1 (2, 9) = 24.36, *P1* = 0.0072, *P2* = 0.0135; F2 (2, 9) = 23.52, *P1* = 0.0314, *P2* = 0.0043; Supplementary Fig. 2b, *n* = 4, F1 (2, 9) = 29.28, *P1* = 0.0043, *P2* = 0.0079; F2 (2, 9) = 0.9, *P* = 0.7857). We further counted the number of TH-positive neurons in the substantia nigra, and quantified the densitometry of TH-positive termini in the striatum. We found that the bilateral substantia nigra and striatum in mice injected with mixed fibrils contained much fewer TH-positive neurons and termini compared to that in mice injected with pure α-synuclein fibrils (Fig. 2b–d; Fig. 2c, *n* = 5, F1 (2, 12) = 13.18, *P1* = 0.0255, *P2* = 0.0485; F2 (2, 12) = 14.84, *P* = 0.0352; Fig. 2d, *n* = 4, F1 (2, 9) = 56.44, *P1* < 0.0001, *P2* = 0.0248; F2 (2, 9) = 63.62, *P1* = 0.0006, *P2* = 0.0003). Furthermore, immunofluorescence staining revealed that α-synuclein fibrils increased the density of microglia compared with the control group, indicating the fibrils triggered neuroinflammation, another pathological characterizes of PD. The mixed fibrils induced more severe microglia activation (Fig. 2e, f; Fig. 2f, *n* = 5, F (2, 12) = 19.61, *P1* = 0.0057, *P2* = 0.0285). These results suggest that the mixed fibrils possess stronger seeding ability and are more prone to transmission than pure α-synuclein fibrils in vivo.

### Overexpression of cofilin 1 promotes the propagation of α-synuclein pathology in vivo

**Fig. 3:**
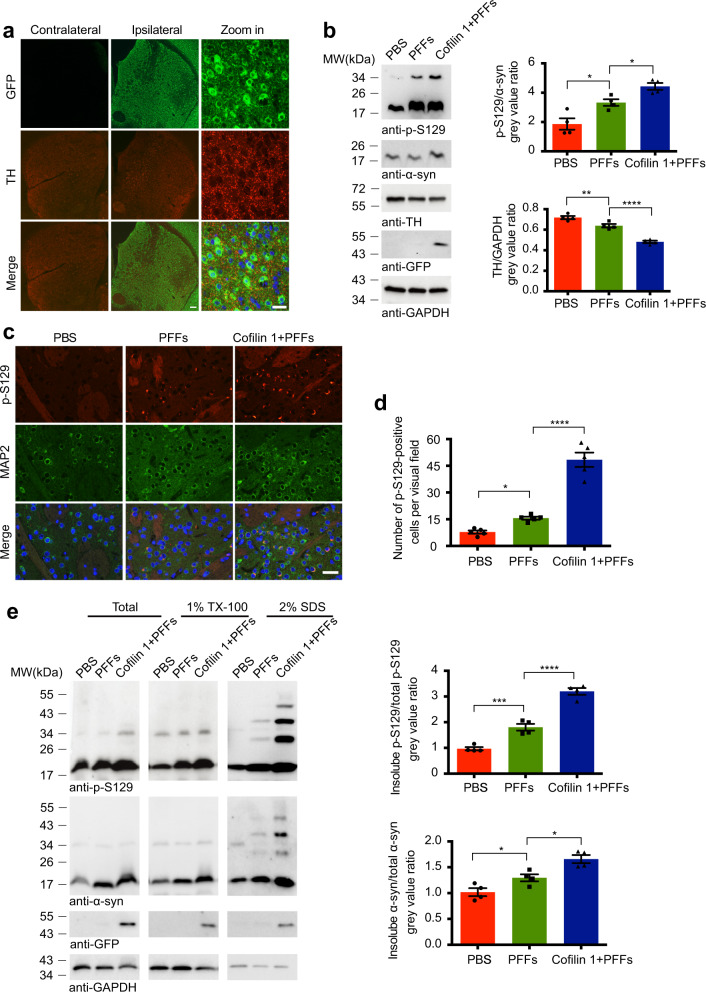
Overexpression of cofilin 1 promotes α-synuclein aggregation in vivo. **a** Immunofluorescence showing the expression of GFP-cofilin 1 in the striatum. Scale bar, 100 μm; Zoom in Scale bar, 20 μm. **b** Western blot detection of TH, total α-synuclein and p-S129 α-synuclein in the striatum of the mouse brain. Data are mean ± SEM; *n* = 4; *P < 0.05, **P < 0.01, ****P < 0.0001 by one-way ANOVA. **c**, **d** Immunofluorescence images showing the expression of p-S129 α-synuclein in the striatum. Scale bar, 20 μm. Data are mean ± SEM; *n* = 5; *P < 0.05, ****P < 0.0001 by one-way ANOVA. **e** Western blot showing the aggregation of α-synuclein into higher-molecular-weight species in the right striatum of mice, especially when cofilin 1 was overexpressed. Data are mean ± SEM; *n* = 4; *P < 0.05, ***P < 0.001, ****P < 0.0001 by one-way ANOVA.

We previously found that the expression of cofilin 1 is increased in brain tissues from patients with PD^9^. Here we further investigated whether overexpression of cofilin 1 in the brain promotes the α-synuclein pathology in a mouse model of PD. The mice were injected with α-synuclein fibrils, together with adeno-associated virus (AAVs) encoding GFP-cofilin 1. Immunofluorescence staining found that the expression of GFP-cofilin 1 was restricted to the right (ipsilateral) striatum (Fig. 3a), but not in the olfactory bulb, hippocampus, and cerebellum (Supplementary Fig. 3a). The neurons in the right striatum express abundant GFP-cofilin 1, which colocalized with p-S129 α-synuclein (Supplementary Fig. 3b). In the SN, GFP-cofilin 1 was only found in the neuronal terminals, but not in the cell bodies of dopaminergic neurons (Supplementary Fig. 3c). When AAV-GFP-cofilin 1 was injected into the striatum without α-synuclein fibrils, the levels of p-S129 were similar to that in the control group both in the striatum and the SN (Supplementary Fig. 3d; *n* = 10; *t* = 0, *P1* > 0.9999; *t* = 0.38, *P2* = 0.7084). When compared with mice injected with only α-synuclein fibrils, injection of AAV-GFP together with α-synuclein fibrils did not alter the levels of p-S129 in the SN and striatum (Supplementary Fig. 3e; *n* = 10; *t* = 0.07785, *P1* = 0.9388; *t* = 0.128, *P2* = 0.8995). Thus, we used mice injected with α-synuclein fibrils as controls to further explore the effect of cofilin 1 on the aggregation and spreading of α-synuclein pathology.

Consistent with the previous reports, immunoblotting showed that the injection of α-synuclein fibrils induced the phosphorylation of endogenous α-synuclein in the striatum. Interestingly, overexpression of cofilin 1 enhanced the phosphorylation of α-synuclein. Furthermore, the expression of TH in the striatum was decreased in mice injected with α-synuclein fibrils when compared with the control mice, indicating the degeneration of dopaminergic neurons. The decrease of TH was exacerbated by cofilin 1 overexpression (Fig. 3b; for p-S129, *n* = 4, F (2, 9) = 19.29, *P1* = 0.0127, *P2* = 0.0262; for TH, *n* = 4, F (2, 9) = 63.35, *P1* = 0.0049, *P2* < 0.0001). Immunofluorescence confirmed that the levels of p-S129 is higher in the striatal neurons when cofilin 1 were overexpressed (Fig. 3c, d; Fig. 3d, *n* = 5, F (2, 12) = 79.75, *P1* = 0.0412, *P2* < 0.0001). Moreover, injection with α-synuclein fibrils induced the formation of higher-molecular-weight species in brain lysates of the right striatum, which was more abundant in mice overexpressing cofilin 1 (Fig. 3e; for insoluble p-S129, *n* = 4, F (2, 9) = 97.54, *P1* = 0.0006, *P2* < 0.0001; for insoluble α-synuclein, *n* = 4, F (2, 9) = 18.6, *P1* = 0.0271, *P2* = 0.0146). These results indicate that cofilin 1 promotes the phosphorylation and aggregation of α-synuclein in the mouse brain.

**Fig. 4:**
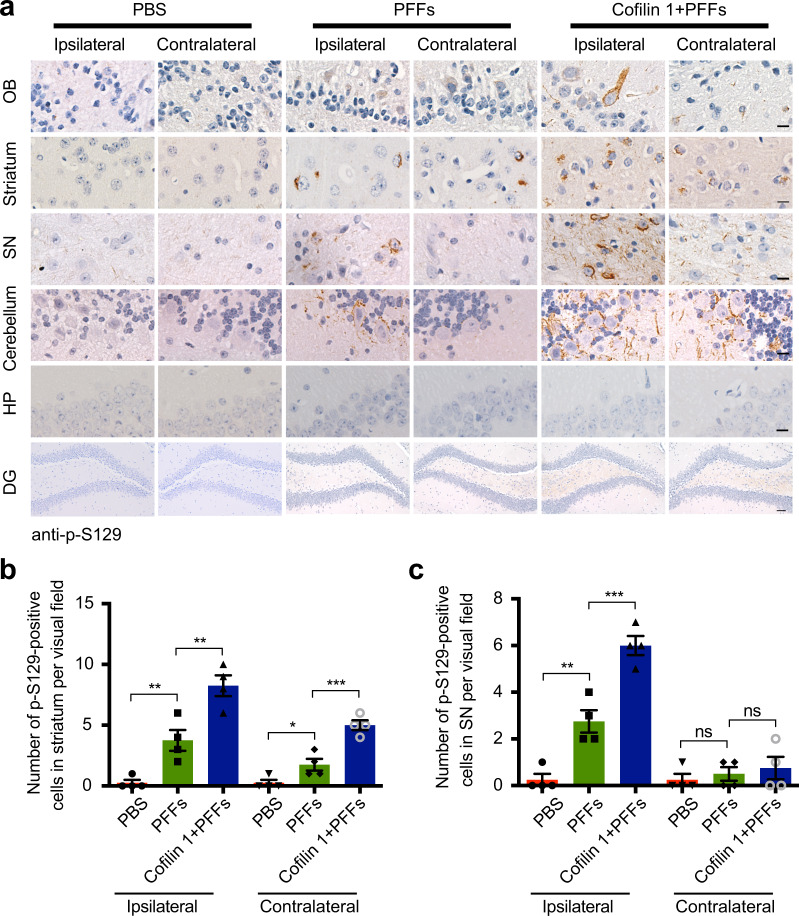
Cofilin 1 accelerates the transmission of α-synuclein aggregates in the mouse brain. **a**–**c** Immunohistochemistry showing the expression of p-S129 α-synuclein in different brain regions of mice injected with PBS, α-synuclein PFFs, and α-synuclein PFFs together with AAV-cofilin 1. Scale bar in DG area, 50 μm. Scale bars in other areas, 20 μm. Bar graph, quantification of p-S129-positive cells in the striatum (**b**) and SN (**c**) of mice. Data are mean ± SEM; *n* = 4; *P < 0.05, **P < 0.01, ***P < 0.001, ns: not statistically significant by one-way ANOVA.

To test the effect of cofilin 1 on the transmission of α-synuclein pathology in the mouse brain, we injected AAV-GFP and AAV-GFP-cofilin 1 in the presence or absence of α-synuclein fibrils into the right striatum of wild-type mice, and compared the expression of p-S129 in different brain regions. We found that AAV-cofilin 1 alone did not induce the phosphorylation of α-synuclein in wild-type mice. AAV-GFP did not affect the levels of p-S129 induced by α-synuclein fibrils (Supplementary Fig. 4a–e; Supplementary Fig. 4b, *n* = 4, *t1* = 0.6547, *P1* = 0.5370; *t2* = 0, *P2* > 0.9999; Supplementary Fig. 4c, *n* = 4, *t* = 0.6547, *P* = 0.5370; Supplementary Fig. 4d, *n* = 4, *t1* = 0.4472, *P1* = 0.6704; *t2* = 0.3974, *P2* = 0.7049; Supplementary Fig. 4e, *n* = 4, *t1* = 1.414, *P1* = 0.2070; *t2* = 0.6547, *P2* = 0.5370). However, overexpression of cofilin 1 in mice injected with α-synuclein fibrils promoted the spreading of α-synuclein pathology in the OB, striatum, SN, and cerebellum of wild-type mice, but not in the hippocampus and DG area (Fig. 4a–c; Fig. 4b, *n* = 4, F1 (2, 9) = 31.73, *P1* = 0.007, *P2* = 0.0031; F2 (2, 9) = 38.59, *P1* = 0.0239, *P2* = 0.0005; Fig. 4c, *n* = 4, F1 (2, 9) = 54.41, *P1* = 0.0014, *P2* = 0.0005; F2 (2, 9) = 0.5, *P* = 0.8731). These results indicate that cofilin 1 enhances the transmission of α-synuclein aggregates in the mouse brain.

### Cofilin 1 enhances dopaminergic neuronal injury induced by α-synuclein fibrils

**Fig. 5:**
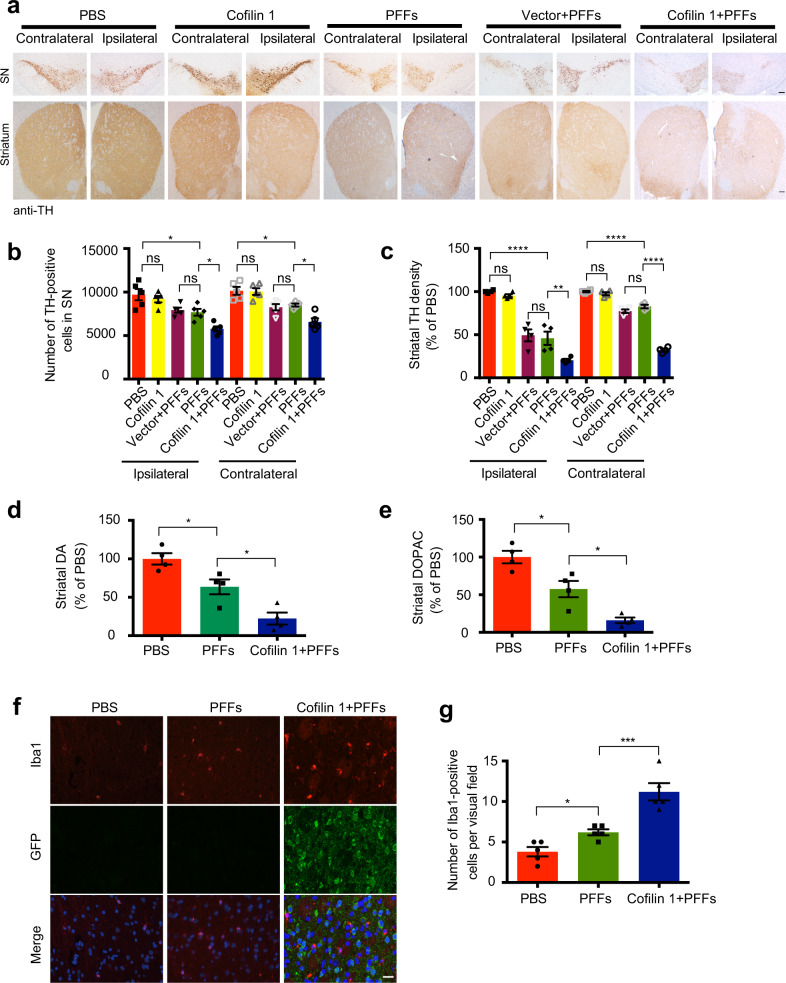
Cofilin 1 promotes the degeneration of dopaminergic neurons induced by α-synuclein fibrils in vivo. **a**–**c** TH immunostaining of the striatum and substantia nigra (SN). The number of TH-positive neurons in the SN and the density of striatal dopaminergic terminals were quantified. Scale bars, 100 μm. Data are mean ± SEM; *n* = 4–5; *P < 0.05, **P < 0.01, ****P < 0.0001, ns: not statistically significant by one-way ANOVA. **d**, **e** Concentrations of DA and DOPAC in the striatal tissues, as determined by HPLC. Data are mean ± SEM; *n* = 4 mice per group; *P < 0.05 by one-way ANOVA. **f**, **g** Cofilin 1 promotes microglia activation. Immunostaining showing the Iba1-positive microglia in the striatum. Scale bar, 20 μm. Data are mean ± SEM; *n* = 5; *P < 0.05, ***P < 0.001 by one-way ANOVA.

Since cofilin 1 accelerates the transmission of α-synuclein inclusions, we further tested whether overexpression of cofilin 1 affects the degeneration of dopaminergic neurons and activation of microglia in vivo. The number of TH-positive neurons and the density of TH-positive terminals in the bilateral substantia nigra and striatum were decreased in the presence of α-synuclein fibrils. The pathology was more severe when cofilin 1 was overexpressed, while AAV-cofilin 1 alone had no effect on the expression of TH compared with the control group (Fig. 5a–c, Fig. 5b, *n* = 5, F1 (4, 20) = 13.42, *P1* = 0.0189, *P2* = 0.0249; F2 (4, 20) = 16.08, *P1* = 0.0207, *P2* = 0.011; Fig. 5c, *n* = 4, F1 (4, 15) = 51.36, *P1* < 0.0001, *P2* = 0.0056; F2 (4, 15) = 245.2, *P* < 0.0001). HPLC analysis of the striatal tissues showed significantly decreased DA and DOPAC concentrations in mice injected α-synuclein fibrils. The overexpression of cofilin 1 with α-synuclein fibrils further decreased the content of DA and DOPAC (Fig. 5d, e; Fig. 5d, *n* = 4, F (2, 9) = 21.7, *P* = 0.0134; Fig. 5e, *n* = 4, F (2, 9) = 26.41, *P* = 0.0102). Hence, cofilin 1 promotes the degeneration of nigrostriatal dopaminergic pathway induced by α-synuclein fibrils. In addition, injection of α-synuclein fibrils induced the activity of microglia, which was further enhanced when cofilin 1 was overexpressed with α-synuclein fibrils (Fig. 5f, g; Fig. 5g, *n* = 5, F (2, 12) = 26.4, *P1* = 0.0395, *P2* = 0.0009). Immunostaining of TH and Iba1 further verified the activation of microglia and the decrease of dopaminergic terminals in the striatum (Supplementary Fig. 5; *n* = 4, F1 (2, 9) = 18.94, *P1* = 0.0148, *P2* = 0.0242; F2 (2, 9) = 34.88, *P1* = 0.015, *P2* = 0.0011). These results indicate that cofilin 1 promotes dopaminergic degeneration and microglial activation in vivo.

### Cofilin 1 promotes behavioral impairments caused by α-synuclein fibrils

**Fig. 6:**
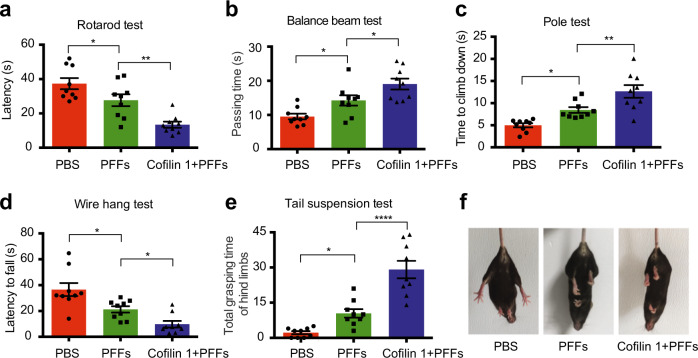
Cofilin 1 enhances behavioral impairments induced by α-synuclein fibrils. Behavioral performance. The behavioral impairments were more severe when cofilin 1 was overexpressed with α-synuclein fibrils, as demonstrated by rotarod test (**a**), balance beam test (**b**), pole test (**c**), wire hang test (**d**), and tail suspension test (**e**). Data are mean ± SEM; *n* = 9 mice per group; *P < 0.05, **P < 0.01, ****P < 0.0001 by one-way ANOVA. (**f**) Representative images showing the hindlimbs clasp in tail suspension test.

To investigate the effect of cofilin 1 on motor dysfunction induced by α-synuclein fibrils, we performed a panel of behavioral tests. Compared to α-synuclein fibrils alone, mice injected with α-synuclein fibrils together with AAV-cofilin 1 showed decreased latency in the rotarod test and wire hang test (Fig. 6a, d; Fig. 6a, *n* = 9 mice per group, F (2, 24) = 16.93, *P1* = 0.0279, *P2* = 0.0042; Fig. 6d, *n* = 9 mice per group, F (2, 24) = 14.37, *P1* = 0.0108, *P2* = 0.0315), indicating that cofilin 1 facilitates the detrimental effect of α-synuclein fibrils on motor function. In agreement with this finding, overexpression of cofilin 1 produced increased passing time in balance beam test and pole test (Fig. 6b, c; Fig. 6b, *n* = 9 mice per group, F (2, 24) = 12.38, *P* = 0.0378; Fig. 6c, *n* = 9 mice per group, F (2, 24) = 16.69, *P1* = 0.0155, *P2* = 0.0084). The tail suspension test also demonstrated an evident motor deficit and increased hind limbs impairment in mice overexpressing cofilin 1 (Fig. 6e, f; *n* = 9 mice per group, F (2, 24) = 32.63, *P1* = 0.024, *P2* < 0.0001). Together, these results indicate that cofilin 1 deteriorates the motor impairments in a mouse model of PD.

## Discussion

The initial pathogenesis of PD involves the conversion of soluble α-synuclein into insoluble aggregates. The misfolded α-synuclein in the central nervous system can aggregate and proliferate by recruiting native proteins via templated conformational change. The concept of “strain” has been proposed in recent years, which suggest that different strains of α-synuclein aggregates contribute to the heterogeneity of synucleiopathies^9^. α-Synuclein aggregates in PD patients also make individual differences due to their specific pathology and neurotoxic phenotypes. Thus, clarifying the diversity of different α-synuclein aggregates will facilitate the understanding of disease progression. Here we report that the mixed fibrils composed of cofilin 1 and α-synuclein are more harmful to mice with stronger seeding and transmission capacity compared with pure α-synuclein fibrils. Cofilin 1 enhances the transmission and propagation of α-synuclein fibrils, thus induces more severe dopaminergic degeneration and motor impairments in mice.

**Fig. 7:**
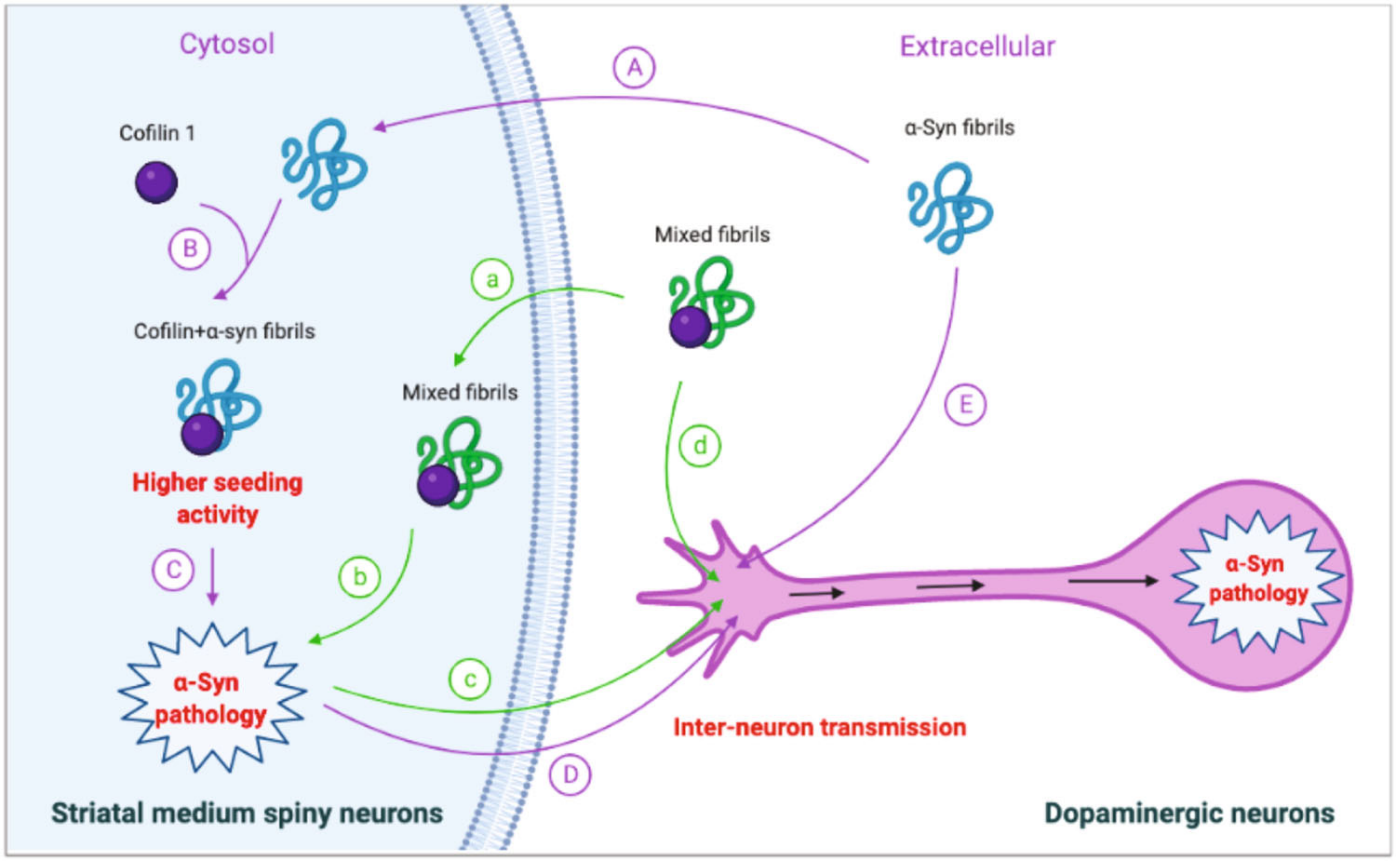
Schematic diagram showing the effect of cofilin 1 in α-syn pathology. Purple lines: overexpression of cofilin 1 in the striatum promotes the spreading of α-syn pathology. (**A**) Injected α-syn fibrils are taken up by the striatal medium spiny neurons. (**B**) Cofilin 1 in the medium spiny neurons interact with α-syn fibrils and enhances its seeding activity. (**C**) α-Syn fibrils seed endogenous α-syn into aggregates. (**D**) Pathogenic α-syn aggregates transfer from the medium spiny neurons to the dopaminergic terminals and spread along the neurites to the SN, inducing pathological α-syn inclusions in dopaminergic neurons. (**E**) α-Syn fibrils are taken up by dopaminergic terminals and spread to dopaminergic neurons in the SN. Green lines: injection of cofilin 1-α-syn mixed fibrils causes α-syn pathology. **a** Cofilin 1-α-syn mixed fibrils are taken up by striatum medium spiny neurons. **b** Mixed fibrils cause α-syn pathology in the striatum. **c** Striatal pathogenic α-syn transmit to dopaminergic neurons in SN, causing neuronal damage. **d** Mixed fibrils are directly transferred to SN through the terminals of dopaminergic neurons.

Here we used a mouse model of synucleinopathy to test the effect of cofilin 1 on the spreading of α-synuclein pathology. Injection of pre-formed α-synuclein fibrils into the right striatum triggered α-synuclein pathology in the striatum, substantia nigra, cerebellum, and OB regions. Overexpression of exogenous GFP-cofilin 1 or injection of cofilin 1-α-syn mixed fibrils in the right striatum accelerated the spreading of α-synuclein pathology to other brain areas and the degeneration of dopaminergic neurons (Fig. 7). This is consistent with our previous report that α-synuclein forms fibrils that are more toxic and prone to propagate in the presence of cofilin 1^17^. Here we also found that the cofilin 1-α-synuclein mixed fibrils are more prone to spread in vivo (Figs. 1, 2). Therefore, we believe the accelerated degeneration of dopaminergic neurons in SN after striatal injection of AAV-cofilin 1 and α-synuclein fibrils is caused by the accelerated transmission of more pathogenic α-synuclein fibrils. The propagation of protein aggregates involves the release of fibrils from the donor cells and the entry into recipient cells. It is generally believed that α-synuclein can be transferred by classic exocytosis and endocytosis pathways (including micropinocytosis), tunneling nanotubes, synapses or synapse-like structures, and receptors^18–20^. Cofilin 1, as a widely expressed actin-binding protein, mediates multiple cell functions including cell migration, vesicle release, and nutrient transport, etc. We found that cofilin 1 not only promoted the propagation of α-synuclein pathology, but also induced more severe dopaminergic neuronal injury and motor impairments.

Cofilin 1 may promote the transmission of α-synuclein pathology between cells through several pathways. Firstly, it may involve the exosome-associated mechanisms. Exosomes derived from neurons contain misfolded α-synuclein, and can transfer between cells, thus mediating the transmission of α-synuclein pathology^21^. A recent study found that the quantity of α-synuclein within these exosomes to be significantly higher in PD patients as compared to controls and correlated with disease severity^22^. Uptake of exosomes loaded with α-synuclein has been shown to induce neuronal cell death^23^. Interestingly, cofilin 1 is one of the major components of exosomes. Activated cofilin 1 can mediate the depolymerization of actin and promote the formation of exosomes^24,25^. It is conceivable that cofilin 1-mediated exosomes may be involved in the spread of α-synuclein pathology in PD. Secondly, cofilin 1 may facilitate the transmission of α-synuclein pathology via vesicle-mediated exocytosis^26^. Some studies found that the release of pathological α-synuclein is non-classical ER-Golgi–independent exocytosis^27^. The released extracellular α-synuclein aggregates can be endocytosed into adjacent cells and finally spread throughout the brain. Cofilin 1 also controls the release and ingestion of vesicles by regulating the depolymerization of actin. It implies that cofilin 1-mediated exocytosis and endocytosis of α-synuclein may also participate in the transmission of α-synuclein pathology. A recent study showed that diverse protein aggregates, including α-synuclein aggregates, exploit the cofilin 1 signaling pathway to overcome the actin barrier and invade cells through endocytosis^28^, just like viruses do. Thirdly, necrotic neuronal death releases intracellular contents into the extracellular space^29^. It has been demonstrated that α-synuclein inclusions can impair autophagosome clearance and disturb the degradation pathway, which finally contributes to more extracellular α-synuclein aggregates^30^. In our study, we found that cofilin 1 enhances the toxicity of α-synuclein aggregates. Therefore, the intracellular α-synuclein aggregates are more prone to be released by damaged neurons, and spread all over the brain. Ultimately, the aggregates act as amplification seeds and finally induce the pathology of PD.

In conclusion, our results indicate that cofilin 1 is an important regulator during the formation of α-synuclein inclusions. It co-aggregates with α-synuclein and form more toxic aggregates, which spreads in the mouse brain, and accelerates the degeneration of dopaminergic neurons. Blocking the cofilin 1 pathway may help to relieve the spreading of α-synuclein pathology in PD.

## Methods

### Mice

Female C57BL/6J mice were used in this project. The mice were housed in standard cages with free access to food and water. Animal care and handling were performed according to the Declaration of Helsinki and guidelines of Renmin Hospital, Wuhan University. The protocol was reviewed and approved by the Animal Care and Use Committee of Renmin Hospital of Wuhan University (No. 20190813). 12-week-old mice received surgical operation. The sample size was determined by Power and Precision (Biostat). Animals were randomly assigned to different groups, and the investigators were blind to the group assignment during the animal experiments.

### Protein purification and preparation of fibrils

His-tagged α-synuclein and cofilin 1 were purified from E. coli through Ni-chelating affinity chromatography and eluted at around 125 mM imidazole. The purity was above 90% as illustrated by SDS-PAGE gel analysis. Protein concentrations were determined by BCA assay (Thermo Fisher). α-Synuclein aggregation was induced by incubating protein at 37 °C with 1000 rpm shaking for 5 days. α-Synuclein fibrillization with or without cofilin 1 was confirmed using the thioflavin T fluorescence assay. Briefly, aliquots of 5 μl incubation samples were diluted to 100 μl with 25 mM thioflavin T in PBS, and tested at 450 nm excitation and 510 nm emission using the SpectraMax plate reader. The verified α-synuclein aggregates were then sonicated for 30 s with 0.5 s pulse on/off (Sonics Vibra cell), aliquoted, snap-frozen in liquid nitrogen, and finally stored at −80 °C.

### Immuno-labeling negative staining electron microscopy

Samples were absorbed onto 300 mesh carbon-coated copper grids for 5 min and were washed by overlaying the grids on a drop of PBS. The grids were blocked with 1% serum bovine albumin (BSA) in PBS for 10 min and sequentially incubated with each primary antibody diluted in 1% BSA/PBS. Following washes with PBS, the grids were sequentially incubated with secondary antibodies (either anti-mouse or anti-rabbit) conjugated to 4 nm or 10 nm colloidal gold, respectively. After washing with PBS, fibrils were negative stained with 1% uranyl acetate in water and grids were dried. Samples were analyzed with electron microscopy and images were obtained with a digital camera. Syn 211 is mouse anti-α-syn monoclonal antibodies (Thermo Fisher Scientific, MA5-12272, 1:200), Cofilin 1 antibody is a rabbit monoclonal antibody (Cell Signaling Technology, 5175S, 1:200).

### AAV packaging and stereotaxic injection

The AAV vector (rAAV-hSyn-EGFP-3XFlag-WPRE-hGH-pA) was purchased from Brain VTA Technology (Wuhan, China). Cofilin 1 was cloned into this vector to express GFP-cofilin 1 under the control of the human synapsin I promoter. Three-month-old wild-type C57BL/6J mice were anesthetized with chloral hydrate. The unilateral intracerebral injection was performed stereotaxically at coordinates anteroposterior (AP) + 0.2 mm and mediolateral (ML)−2.0 mm relative to the bregma, and dorsoventral (DV)−2.7 mm from the dural surface. 5 μg sonicated fibrils (1 μg/μl) with or without 300 nl AAV-cofilin 1 were injected into each site with a 10-μl glass syringe (Hamilton, NV) with a fixed needle at a rate of 0.25 μl/min. The needle remained in place for 5 min before it was removed slowly (over 2 min).

### Western blot analysis

The mouse brain tissue samples were lysed in lysis buffer (50 mM Tris, pH 7.4, 40 mM NaCl, 1 mM EDTA, 0.5% Triton X-100, 1.5 mM Na3VO4, 50 mM NaF, 10 mM sodium pyrophosphate and 10 mM sodium β-glycerophosphate, supplemented with protease inhibitors cocktail), and centrifuged for 15 min at 16,000 *g*. The supernatant was boiled in SDS loading buffer. After SDS-PAGE, the samples were transferred to a nitrocellulose membrane. Primary antibodies to the following targets were used: GAPDH (Proteintech, 60004-1-Ig, 1:8000), TH (Sigma-Aldrich, AB152, 1:1000), p-Ser129 α-synuclein (Cell Signaling Technology, 23706 S, 1: 1000), Cofilin 1 (Cell Signaling Technology, 5175 S, 1:1000). The membranes were incubated with primary antibodies overnight at 4 °C. The membranes were washed 3 times in PBST and incubated with HRP-conjugated secondary antibodies. The signals were developed using enhanced chemiluminescent (ECL) substrates. The experiment was repeated at least three times. All blots derived from the same experiment and were processed in parallel.

### Triton-X and SDS fractionation of soluble and insoluble α-synuclein

For sequential extraction of soluble and insoluble α-synuclein, the brain samples were incubated in lysis buffer containing 1% Triton X-100 (TX-100) and protease and phosphatase inhibitors. After sonication using a fine probe [0.5 s pulse at an amplitude of 20%, 10 times (Ningbo Toshiba Ultrasonic Cell Crusher JY99-IIDN, China)], brain lysates were incubated on ice for 30 min. The samples were taken as total fraction. Then the samples were centrifuged at 100,000 *g* for 30 min at 4 °C. The supernatant (TX-100 soluble fraction) was collected while the pellet was washed in 1% Triton X-100, sonicated as described above, and then centrifuged for another 30 min at 100,000 *g*. The supernatant was discarded whereas the pellet (TX-100 insoluble fraction) was resuspended in 2% sodium dodecyl sulfate (SDS) supplemented with protease and phosphatase inhibitor, and sonicated using a fine probe (0.5 s pulse at amplitude of 20%, 15 times). The samples were lysed at room temperature for 30 min and centrifuged. The supernatant was retained as the SDS fraction.

### Immunostaining

The paraffin slices were dewaxed and treated with 0.3% H_2_O_2_ for 10 min. Then, sections were washed three times in PBS and blocked in 3% BSA for 1 h followed by overnight incubation with corresponding antibodies at 4 °C respectively. To verify the expression of p-Ser129 α-synuclein and TH, the paraffin sections of mouse brains were stained with anti-TH (Sigma-Aldrich, AB152, 1:500) and anti-p-Ser129 α-synuclein antibodies (Biolegend, #825701, 1:500) in immunohistochemistry, the signal was developed using the Histostain-SP kit (Invitrogen). Microglia were identified using the antibody specific for Iba1 (Wako, 019-19741, 1:500) in immunofluorescence. The slides were washed three times in PBS and incubated with Alexa Fluor 488/594-conjugated fluorescent secondary antibodies (Thermo Fisher Scientific, 1:500) for 1 h at room temperature. To detect the colocalization of p-S129 α-synuclein and thioflavin S in mouse brain, the sections were incubated with mouse anti-p-S129 α-synuclein primary antibody (Biolegend, #825701, 1:500) overnight at 4 °C, and then incubated with Alexa Fluor 594-conjugated anti-mouse IgG for 1 h at room temperature. After brief rinse in PBS, the sections were stained for 5 min with 0.5% thioflavin S in 50% ethanol, then washed with 50% ethanol and placed in distilled water. Finally, the slides were covered with a glass cover using the mounting solution and examined under an Olympus inverted microscope (Olympus TH4-200, Japan). The experiment was repeated at least three times.

### HPLC analysis of DA and DOPAC

DA and DOPAC levels were determined by HPLC with coulometric detection. The right striatum of each brain sample was processed individually. Samples were homogenized in 0.1 M HClO_4_ solution and centrifuged at 18,000 g for 15 min at 4 °C. Aliquots of supernatant fractions were filtered, then injected into an Ultrasphere 5 μm ODS column, 250 × 4.6-mm (Hichrom Limited), and separated with a mobile phase. The DA and DOPAC amounts were then quantified by comparison to internal standards.

### Behavioral tests

Behavioral impairments were tested 24 weeks after the fibrils injection with balance beam test, pole test, rotarod test, wire hang test, and tail suspension test. Mice were habituated to the test room for at least 30 mins before each test. The same sets of animals were used for different tests starting with the less aversive test. In order to recover, mice were given at least one day between tests. All behavior apparatuses were cleaned between each trial with 70% ethanol. In the rotarod tests, animals were trained for 2 min at a speed of 4 r.p.m. After this initial training, mice performed eight trials for a maximum of 5 min with increasing speed starting from 4 r.p.m. and increasing to 40 r.p.m. The fall-off time was recorded. In the tail suspension test, mice were held by their tail for 30 s and the frequency and duration of hindlimb clasping was scored. In the balance beam test, mice were placed on one end of a narrow beam (20 mm/12 mm width) suspended 20 cm above a soft mattress, and their movement toward the other end was recorded by a video camera. The number of missteps (paw faults, or slips) during the trip was scored. In wire hang test, which measures muscle strength, was conducted by placing mice on top of a 0.5 cm wire mesh, inverting the mesh, and keeping it suspended at 1 m above the cage for 2 min. Mice that let go will fall into the cage filled with soft bedding material. The time to fall (in seconds) was determined for each mouse. In the pole test, mice were held by their tail and placed on top of the pole (according to the fact that the mouse’s hindlimbs are on the top), the climbing time from the top to the forelimbs touching the bottom of the pole was recorded.

### Stereological quantification of TH-positive cells

The number of TH-positive cells in the substantia nigra was estimated with a random-sampling stereological counting method. For each animal, every fourth section throughout the rostrocaudal extent of the substantia nigra and every fourth section covering the entire extent of the striatum were incorporated into the counting procedure. The investigator was blinded to the conditions of the experiment.

### Statistical analyses

All data were expressed as means ± SEM. Statistical analysis was performed using either Student’s *t*-test (two-group comparison) or one-way ANOVA followed by LSD *post hoc* test (more than two groups), and *P* values < 0.05 were considered significant.

### Reporting summary

Further information on research design is available in the Nature Research Reporting Summary linked to this article.

## Supplementary information

Supplementary Figure and Original scans of WB

Reporting Summary

## Data Availability

The authors declare that the data supporting the findings of this study are available within the article and its supplementary information files. Raw data will be made available upon reasonable request.
