# Supplementary material for: Cofilin 1 promotes the pathogenicity and transmission of pathological α-synuclein in mouse models of Parkinson’s disease
Source: NPJ Parkinsons Dis. 2022 Jan 10;8:1. doi: 10.1038/s41531-021-00272-w (PMC8748615; doi:10.1038/s41531-021-00272-w)

Supplementary Figure 1

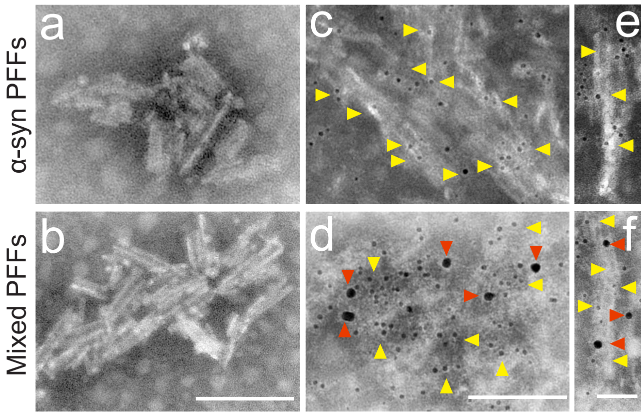

Supplementary Figure 1. The morphology of cofilin 1-α-synuclein mixed fibrils. (a, b) Electron microscopy (EM) showing the formation of α-synuclein fibrils and mixed fibrils. Scale bar, 100 nm. (c, d) Immuno-EM showing the composition of α-synuclein fibrils and mixed fibrils. Shown are the mouse antibody to α-synuclein (4 nm gold, yellow arrowheads) and rabbit antibody to cofilin 1 (10 nm gold, red arrowheads). Scale bar, 100 nm. (e, f) Enlarged individual fibrils of α-synuclein fibrils and mixed fibrils. Scale bar, 50 nm.

Supplementary Figure 2

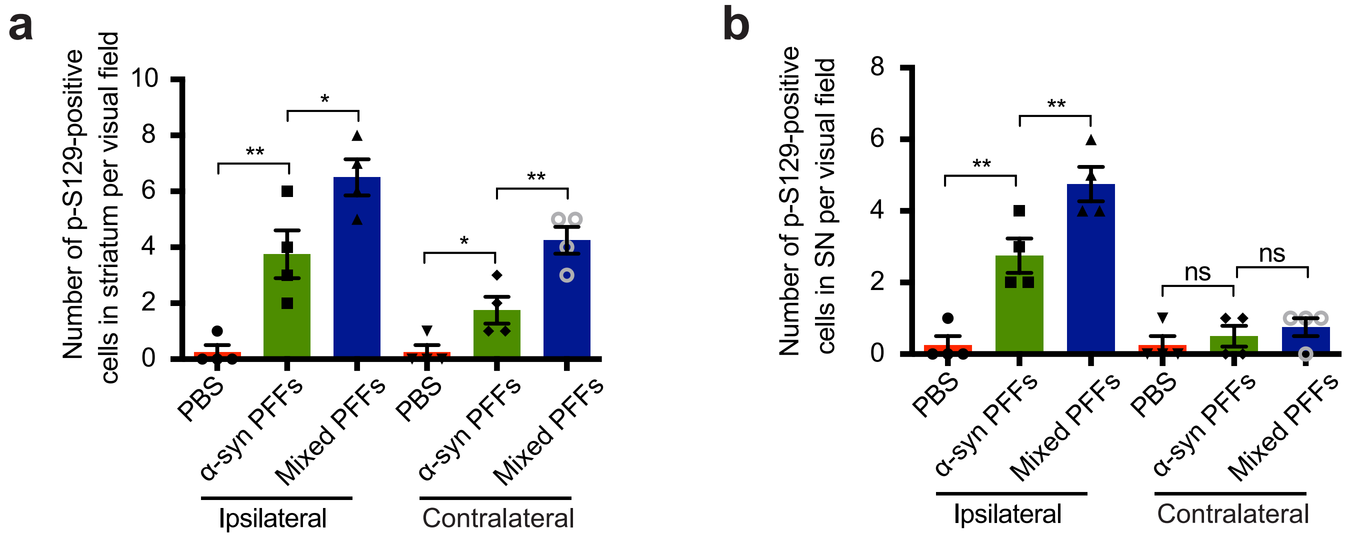

Supplementary Figure 2. The quantification of p-S129-positive cells in the striatum and SN from mice injected with PBS, α-synuclein PFFs, and mixed PFFs. Data are mean ± SEM; n = 4; $*P < 0.05$, $**P < 0.01$, ns: not statistically significant by one-way ANOVA.

Supplementary Figure 3

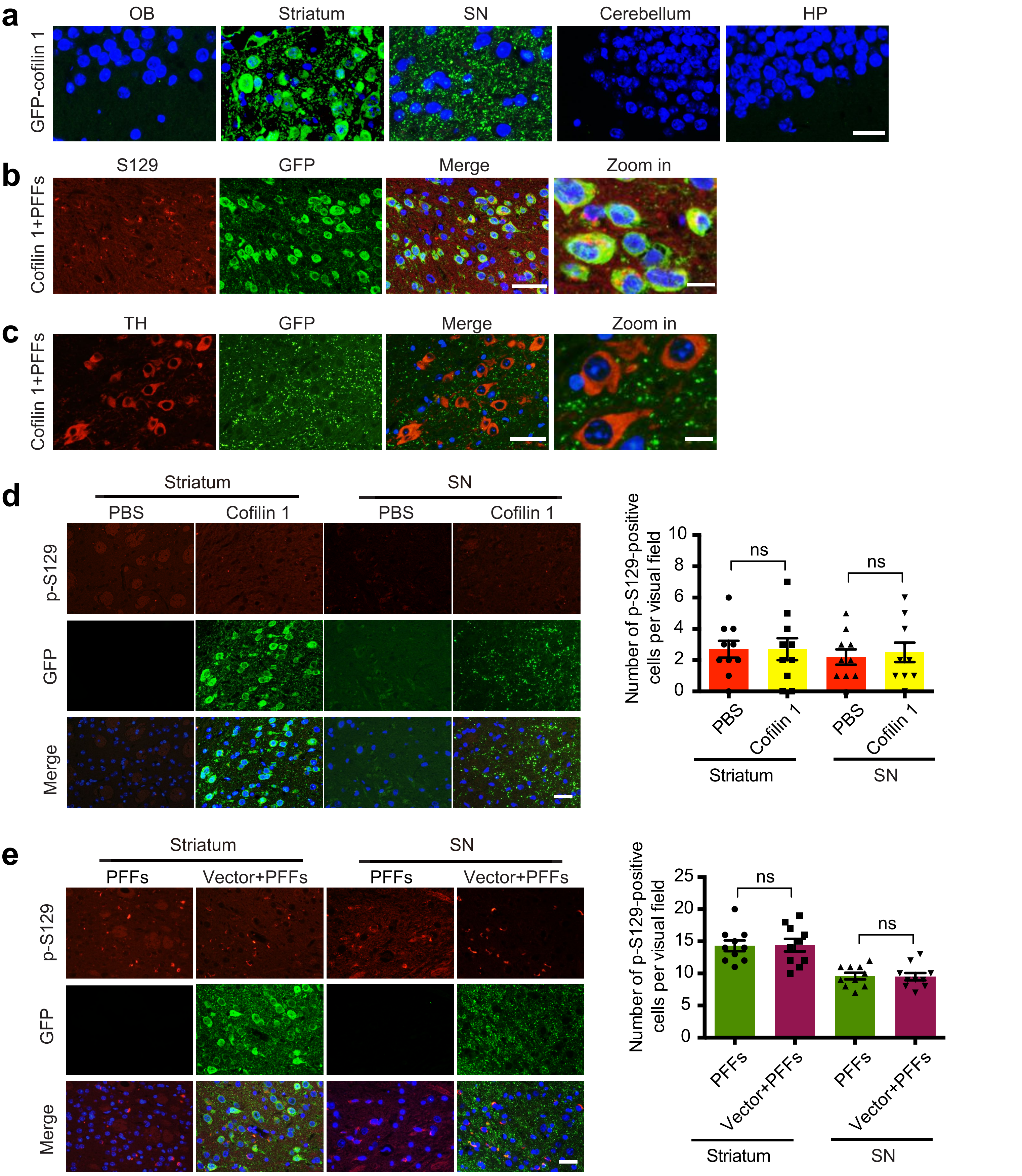

Supplementary Figure 3. The expression pattern of GFP-cofilin 1 and p-S129 α-synuclein. (a) Immunofluorescence images showing the expression of GFP-cofilin 1 in different brain regions of mice injected with α-synuclein fibrils and AAV-cofilin 1, including the right striatum, SN, olfactory bulb, hippocampus, and cerebellum. Scale bar, 20 μm. (b, c) Double immunofluorescence staining of p-S129 α-synuclein and GFP-cofilin 1 in the right striatum (b) and SN (c) of mice injected with α-synuclein fibrils and AAV-cofilin 1. Scale bar, 20 μm. Zoom in scale bar, 5 μm. (d, e) Immunofluorescence images showing the expression of p-S129 α-synuclein in the striatum and SN. Scale bar, 20 μm. Data are mean ± SEM; n = 10; ns: not statistically significant by t-test.

Supplementary Figure 4

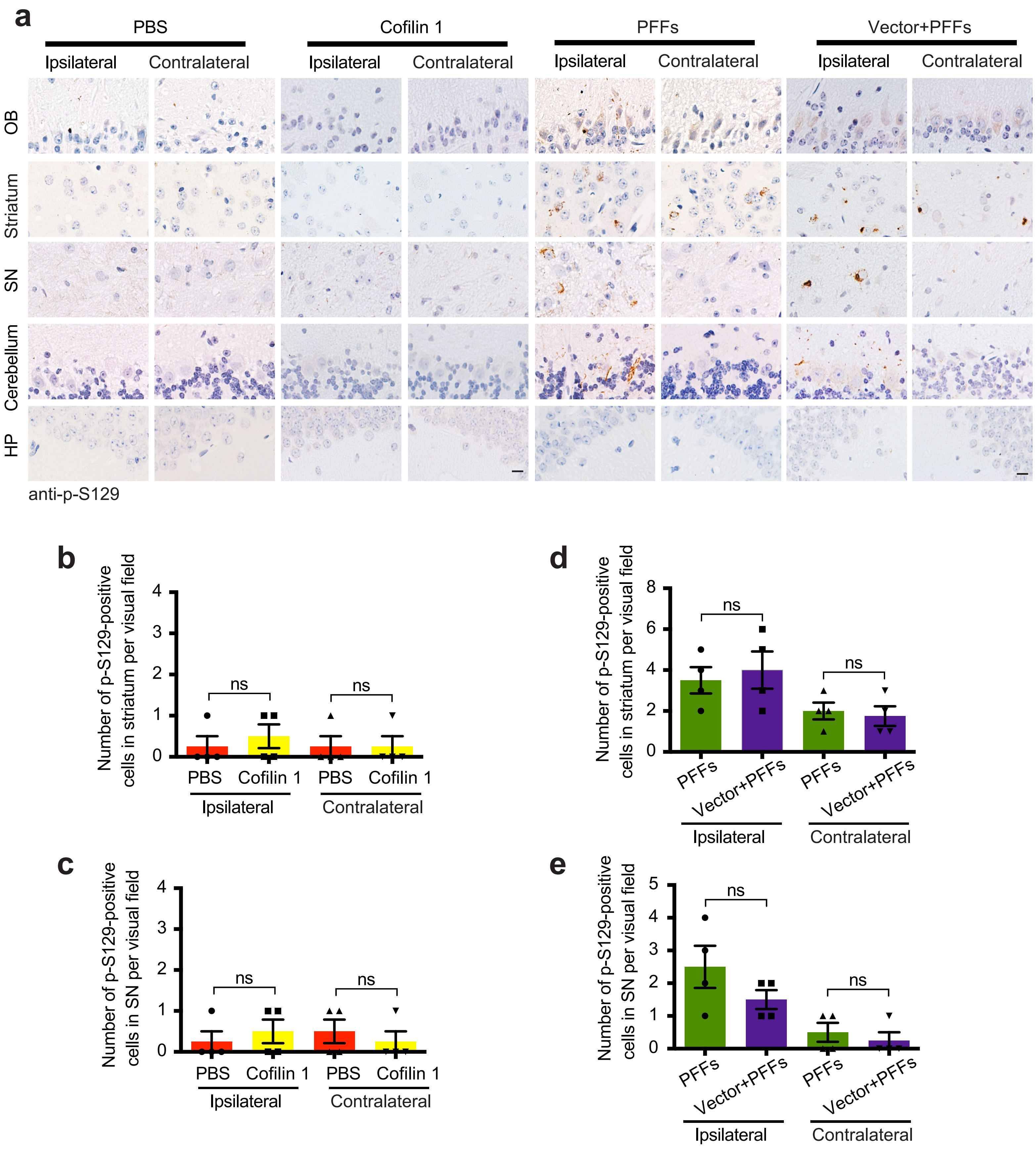

Supplementary Figure 4. The expression of p-S129 α-synuclein in different brain regions of mice injected with PBS, AAV-cofilin 1, α-synuclein PFFs, and α-synuclein PFFs together with AAV-control. Scale bars, 20 μm (a). Bar graph, the quantification of p-S129-positive cells in the striatum and SN from mice injected with PBS, cofilin 1 (b, c), α-synuclein PFFs and α-synuclein PFFs together with AAV-control (d, e). Data are mean ± SEM; n = 4; ns: not statistically significant by one-way ANOVA.

Supplementary Figure 5

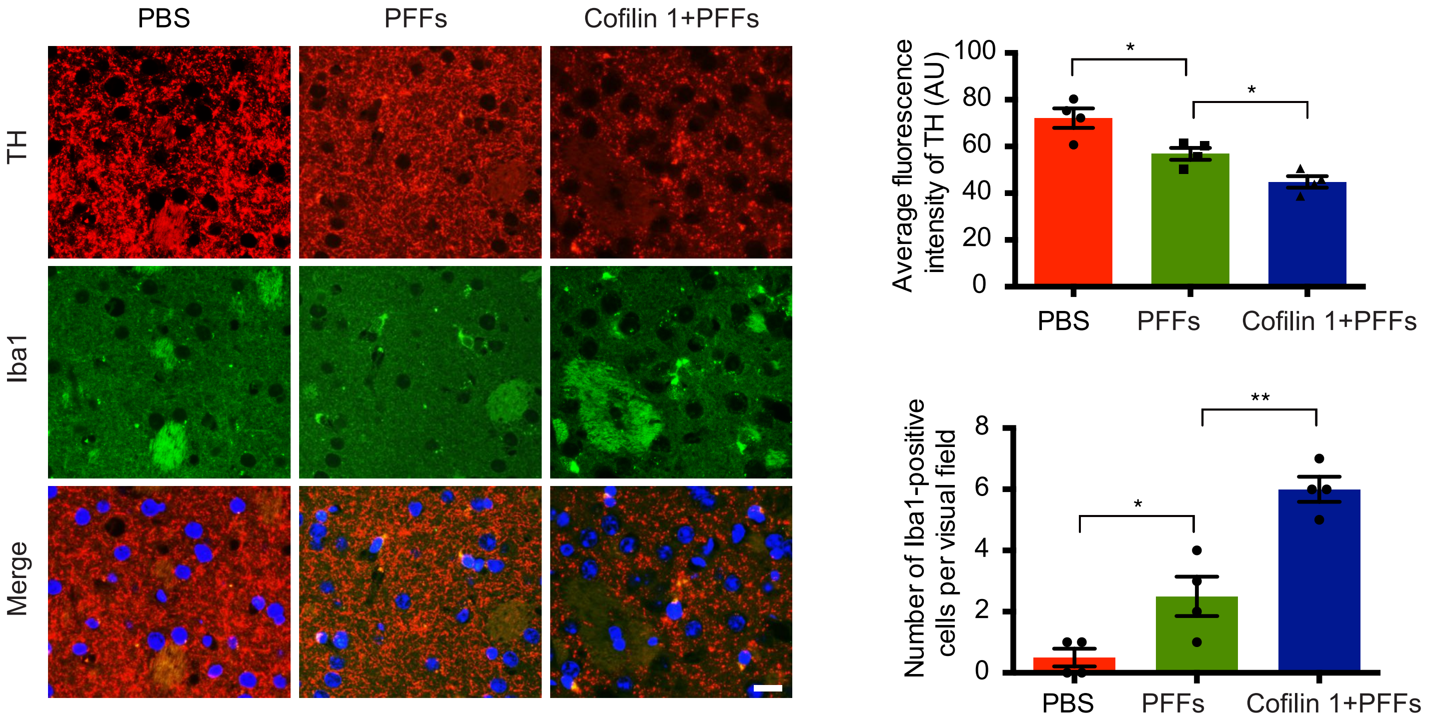

Supplementary Figure 5. Double immunofluorescence of Iba1 and TH in the striatum. Scale bar, 20 μm. Data are mean ± SEM; n = 4; *P < 0.05, **P < 0.01 by one-way ANOVA.

Original scans of WB

Figure 1a

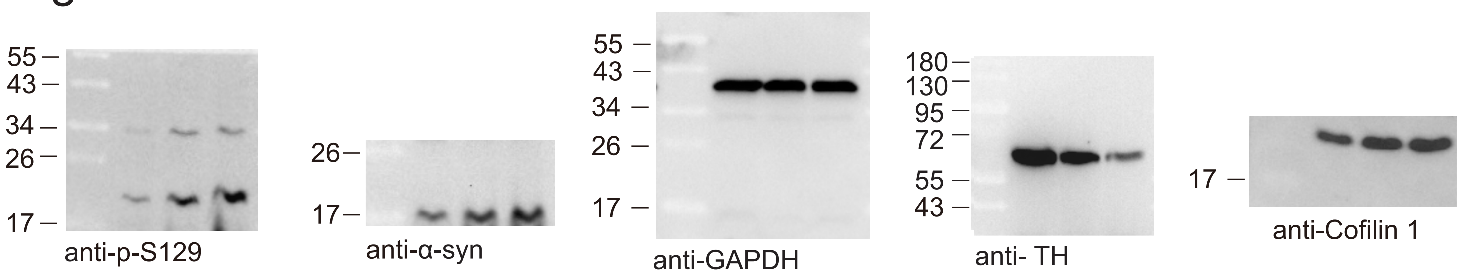

Figure 3b

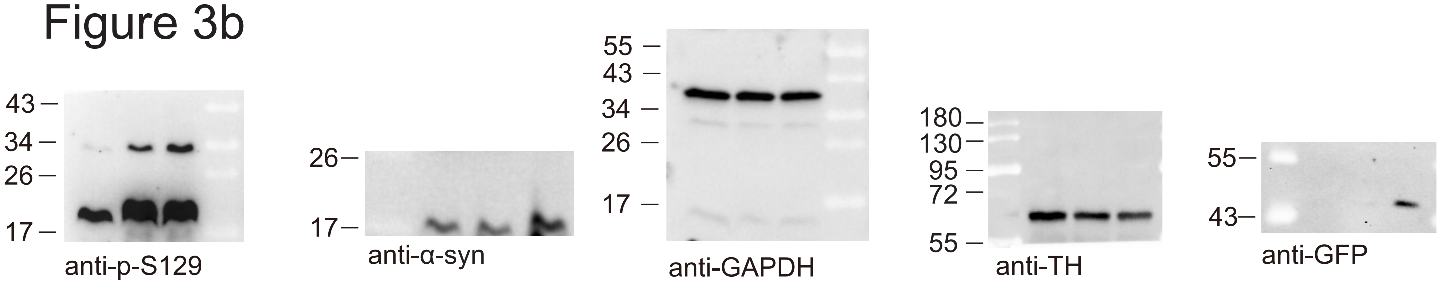

Figure 3e

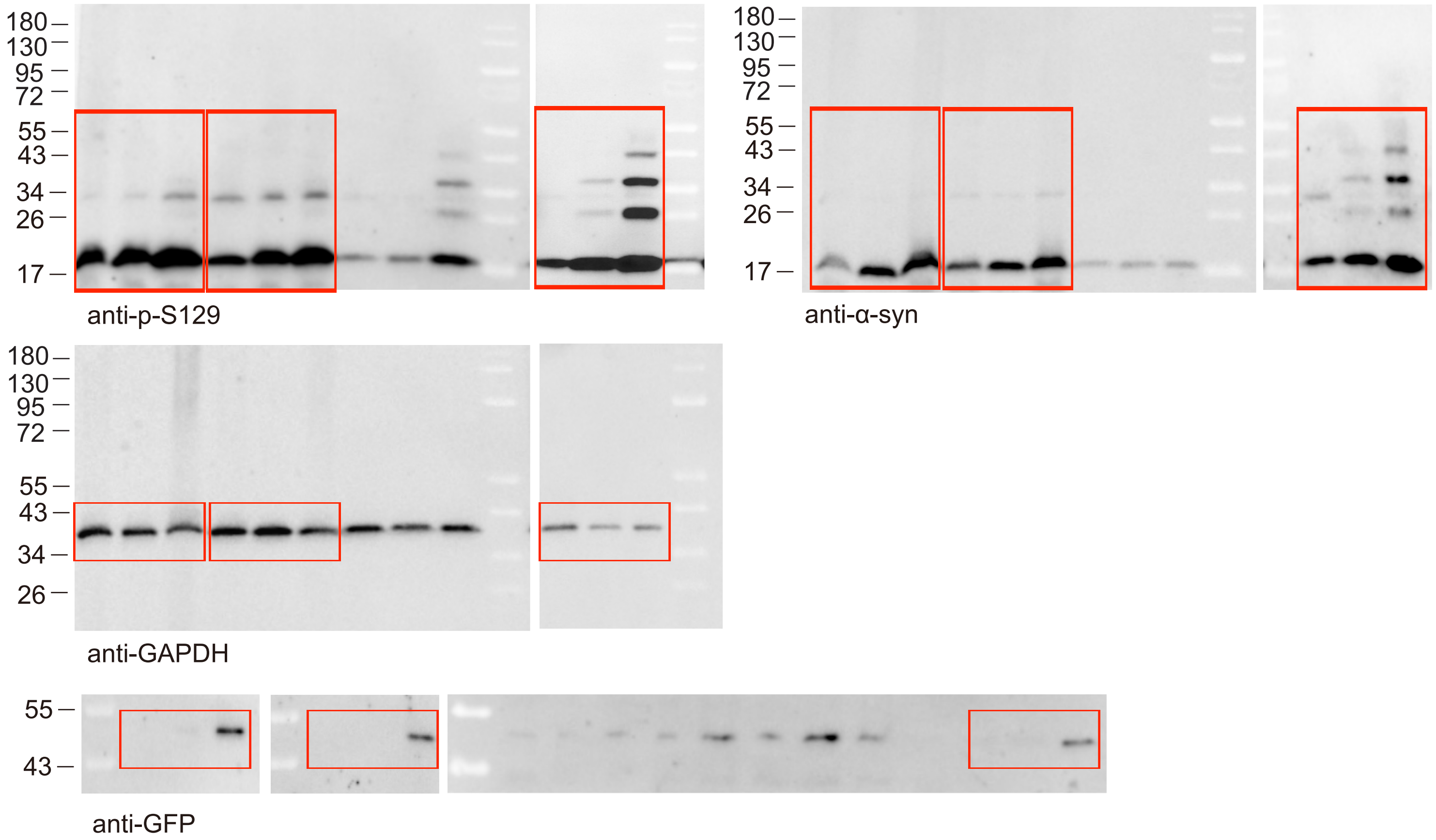

Supplement: Supplementary file 1 — Supplementary Figure and Original scans of WB [file 41531_2021_272_MOESM1_ESM.pdf]
