# Supplementary material for: Cofilin 1 promotes the pathogenicity and transmission of pathological α-synuclein in mouse models of Parkinson’s disease
Source: NPJ Parkinsons Dis. 2022 Jan 10;8:1. doi: 10.1038/s41531-021-00272-w (PMC8748615; doi:10.1038/s41531-021-00272-w)
Supplement: Supplementary file 2 — Reporting Summary [file 41531_2021_272_MOESM2_ESM.pdf]

nature portfolio

Corresponding author(s): Zhentao Zhang

Last updated by author(s): Dec 14, 2021

Reporting Summary

Nature Portfolio wishes to improve the reproducibility of the work that we publish. This form provides structure for consistency and transparency in reporting. For further information on Nature Portfolio policies, see our Editorial Policies and the Editorial Policy Checklist.

Statistics

For all statistical analyses, confirm that the following items are present in the figure legend, table legend, main text, or Methods section.

n/a	Confirmed	
☐	☒	The exact sample size (*n*) for each experimental group/condition, given as a discrete number and unit of measurement
☐	☒	A statement on whether measurements were taken from distinct samples or whether the same sample was measured repeatedly
☐	☒	The statistical test(s) used AND whether they are one- or two-sided<br>*Only common tests should be described solely by name; describe more complex techniques in the Methods section.*
☐	☒	A description of all covariates tested
☐	☒	A description of any assumptions or corrections, such as tests of normality and adjustment for multiple comparisons
☐	☒	A full description of the statistical parameters including central tendency (e.g. means) or other basic estimates (e.g. regression coefficient) AND variation (e.g. standard deviation) or associated estimates of uncertainty (e.g. confidence intervals)
☐	☒	For null hypothesis testing, the test statistic (e.g. *F*, *t*, *r*) with confidence intervals, effect sizes, degrees of freedom and *P* value noted<br>*Give P values as exact values whenever suitable.*
☒	☐	For Bayesian analysis, information on the choice of priors and Markov chain Monte Carlo settings
☒	☐	For hierarchical and complex designs, identification of the appropriate level for tests and full reporting of outcomes
☒	☐	Estimates of effect sizes (e.g. Cohen's *d*, Pearson's *r*), indicating how they were calculated

Our web collection on statistics for biologists contains articles on many of the points above.

Software and code

Policy information about availability of computer code

Data collection	The data came from corresponding HPLC and behavioral software, Immunostaining images were obtained using Olympus inverted microscope (Olympus TH4-200, Japan). The schematic diagram was created in BioRender.com
Data analysis	Graphpad Prism 6.0 and Image J were used for statistics and analysis.

For manuscripts utilizing custom algorithms or software that are central to the research but not yet described in published literature, software must be made available to editors and reviewers. We strongly encourage code deposition in a community repository (e.g. GitHub). See the Nature Portfolio guidelines for submitting code & software for further information.

Data

Policy information about availability of data

All manuscripts must include a data availability statement. This statement should provide the following information, where applicable:

- Accession codes, unique identifiers, or web links for publicly available datasets
- A description of any restrictions on data availability
- For clinical datasets or third party data, please ensure that the statement adheres to our policy

The authors declare that the main data supporting the findings of this study are available within the article and its supplementary information files. Extra data will be made available upon reasonable request.

Field-specific reporting

Please select the one below that is the best fit for your research. If you are not sure, read the appropriate sections before making your selection.

☒ Life sciences ☐ Behavioural & social sciences ☐ Ecological, evolutionary & environmental sciences

For a reference copy of the document with all sections, see nature.com/documents/nr-reporting-summary-flat.pdf

Life sciences study design

All studies must disclose on these points even when the disclosure is negative.

Sample size	The sample size was determined by Power and Precision (Biostat).
Data exclusions	No data was excluded.
Replication	The experiment was repeated at least three times.
Randomization	Animals were randomly assigned to different groups
Blinding	The investigators were blind to the group assignment during the animal experiments.

Reporting for specific materials, systems and methods

We require information from authors about some types of materials, experimental systems and methods used in many studies. Here, indicate whether each material, system or method listed is relevant to your study. If you are not sure if a list item applies to your research, read the appropriate section before selecting a response.

Materials & experimental systems

n/a	Involved in the study	
☐	☒	Antibodies
☒	☐	Eukaryotic cell lines
☒	☐	Palaeontology and archaeology
☐	☒	Animals and other organisms
☒	☐	Human research participants
☒	☐	Clinical data
☒	☐	Dual use research of concern

Methods

n/a	Involved in the study	
☒	☐	ChIP-seq
☒	☐	Flow cytometry
☒	☐	MRI-based neuroimaging

Antibodies

Antibodies used	syn211 (Thermo Fisher Scientific, MA5-12272); Cofilin 1 (Cell Signaling Technology, 5175S); GAPDH (Proteintech, 60004-1-Ig), TH (Sigma-Aldrich, AB152), p-Ser129 α-synuclein (Cell Signaling Technology, 23706S), p-Ser129 α-synuclein (Biolegend, #825701) ,Iba1 (Wako, 019-19741)
Validation	All antibodies were validated by the websites and product pages of vendors.

Animals and other organisms

Policy information about studies involving animals; ARRIVE guidelines recommended for reporting animal research

Laboratory animals	12-week-old female wild-type C57BL/6J mice were used in this project.
Wild animals	The study did not involve wild animals.
Field-collected samples	The study did not involve samples collected from field.
Ethics oversight	The animal protocols and experiments were performed according to the approval of the animal use and care guidelines established by the Laboratory Animal Welfare & Ethics Committee of Renmin Hospital, Wuhan University (Protocol no.20190813).

Note that full information on the approval of the study protocol must also be provided in the manuscript.
